# Investigation of heteroscedasticity in polygenic risk scores across 15 quantitative traits

**DOI:** 10.3389/fgene.2023.1150889

**Published:** 2023-05-09

**Authors:** Hyein Jung, Hae-Un Jung, Eun Ju Baek, Ju Yeon Chung, Shin Young Kwon, Ji-One Kang, Ji Eun Lim, Bermseok Oh

**Affiliations:** ^1^Department of Biomedical Science, Graduate School, Kyung Hee University, Seoul, Republic of Korea; ^2^ Mendel, Seoul, Republic of Korea; ^3^Department of Biochemistry and Molecular Biology, School of Medicine, Kyung Hee University, Seoul, Republic of Korea

**Keywords:** polygenic risk score (PRS), linear regression model, quantitative trait, prediction accuracy, heteroscedasticity

## Abstract

The polygenic risk score (PRS) could be used to stratify individuals with high risk of diseases and predict complex trait of individual in a population. Previous studies developed a PRS-based prediction model using linear regression and evaluated the predictive performance of the model using the *R* ^*2*^ value. One of the key assumptions of linear regression is that the variance of the residual should be constant at each level of the predictor variables, called homoscedasticity. However, some studies show that PRS models exhibit heteroscedasticity between PRS and traits. This study analyzes whether heteroscedasticity exists in PRS models of diverse disease-related traits and, if any, it affects the accuracy of PRS-based prediction in 354,761 Europeans from the UK Biobank. We constructed PRSs for 15 quantitative traits using LDpred2 and estimated the existence of heteroscedasticity between PRSs and 15 traits using three different tests of the Breusch-Pagan (BP) test, score test, and F test. Thirteen out of fifteen traits show significant heteroscedasticity. Further replication using new PRSs from the PGS catalog and independent samples (*N* = 23,620) from the UK Biobank confirmed the heteroscedasticity in ten traits. As a result, ten out of fifteen quantitative traits show statistically significant heteroscedasticity between the PRS and each trait. There was a greater variance of residuals as PRS increased, and the prediction accuracy at each level of PRS tended to decrease as the variance of residuals increased. In conclusion, heteroscedasticity was frequently observed in the PRS-based prediction models of quantitative traits, and the accuracy of the predictive model may differ according to PRS values. Therefore, prediction models using the PRS should be constructed by considering heteroscedasticity.

## Introduction

Genome-wide association studies (GWAS) have identified numerous genetic variants associated with various complex traits (MacArthur et al., 2017). This has enhanced our understanding of the biological pathways and treatment methods for diseases (Visscher et al., 2012; Tam et al., 2019). However, the small effect size of each genetic variant only explained a fraction of the phenotypic variation (Yang et al., 2011). Polygenic risk scores (PRSs) were proposed to apply these genetic variants in the clinical or prevention fields (Lewis and Vassos, 2020). They were constructed by aggregating all the effects of the genetic variants identified by GWASs (Choi et al., 2020). This could potentially be used as a powerful tool to provide personalized medicine (Polderman et al., 2015). The PRS performance has been improved owing to recent large-scale GWAS (Inouye et al., 2018; Duncan et al., 2019) and the development of advanced PRS construction methods (Vilhjalmsson et al., 2015; Prive et al., 2020; Ruan et al., 2022).

Previous studies showed the possibility of PRS to stratify individuals and predict complex traits in large populations (Khera et al., 2019; Ruan et al., 2022; Tanigawa et al., 2022). An estimation of the PRSs for five common diseases showed that the proportion of the population that was at a three-fold increased risk was 8.0% for coronary artery disease (CAD), 6.1% for atrial fibrillation (AF), 3.5% for type 2 diabetes (T2D), 3.2% for inflammatory bowel disease (IBD), and 1.5% for breast cancer (BC) (Khera et al., 2018). Moreover, significant differences were found in the prevalence of obesity [body mass index (BMI) ≥ 30 kg/m^2^] across deciles of PRS for BMI (Khera et al., 2019). Determination of PRSs for over 1,500 traits (including disease outcomes and quantitative traits) in the UK Biobank led to the systemic construction of PRS models and their predictive performance was evaluated: the predictive performance was significantly increased in the 813 PRS models compared to the covariate-only model including age, sex, types of genotyping arrays, and the principal component of genotypes (Tanigawa et al., 2022).

The predictive performance of various PRS models was evaluated by *R* ^2^ (the proportion of variance explained by PRS) for the linear regression model (Vilhjalmsson et al., 2015; Khera et al., 2019; Prive et al., 2020; Ruan et al., 2022; Tanigawa et al., 2022). However, one of the key assumptions of linear regression is that the variance of the residual should be constant at each level of the predictor variables (homoscedasticity) (Hayes and Cai, 2007). Heteroscedasticity (the complementary notion of homoscedasticity) occurs when the residuals at each level of the predictor variable(s) have unequal variances (White, 1980; Andy, 2009). The *R* ^2^ for homoscedasticity was equally estimated at each level of predictor variable(s). However, the *R* ^2^ may not be equally estimated at each level of predictor variables if there is a non-constant variance of residuals (such as heteroscedasticity). The predictive performance for previous PRS models was evaluated without considering whether they are homoscedastic or heteroscedastic (Vilhjalmsson et al., 2015; Khera et al., 2019; Prive et al., 2020; Ruan et al., 2022; Tanigawa et al., 2022). Other studies suggest that PRS models show heteroscedasticity for obesity-related traits (Sulc et al., 2020; Baek et al., 2022). Therefore, PRS models for other traits may also show heteroscedasticity, and it is necessary to test heteroscedasticity in PRS models for various traits.

This study analyzed whether heteroscedasticity exists in PRS models for 15 quantitative traits and whether heteroscedasticity affects the accuracy of PRS-based prediction models using simulation and real data from the UK Biobank. First, we tested whether the difference in the variance of the residuals affects the difference in prediction accuracy under the condition of heteroscedasticity using simulation data. Second, PRSs were constructed for 15 quantitative traits using actual data from the UK Biobank. Heteroscedasticity of the PRS-based prediction models was investigated using three statistical methods: Breusch-Pagan (BP) test, score test, and F test. We then investigated whether heteroscedasticity affected the accuracy of the PRS-based prediction models.

## Materials and methods

### Simulation study

Homoscedasticity describes a situation in which the error variance is constant across all levels of predictor variable(s) in a linear regression model. In other words, the variability of the errors is the same across all values of the independent variable(s) (Hayes and Cai, 2007; Andy, 2009; Astivia and Zumbo, 2019). In contrast, heteroscedasticity describes a situation in which the error variance is not constant across all levels of the predictor variable(s) in linear regression model. In this case, the variability of the errors is different across all values of the independent variable(s).

To understand heteroscedasticity and homoscedasticity, we generated twelve sets of simulation data: 1) homoscedastic data (HS0) with a *R* ^2^ value of 0.9, 2) HS0 with a *R* ^2^ value of 0.5, 3) HS0 with a *R* ^2^ value of 0.1, 4) mildly heteroscedastic data (HS1) with a *R* ^2^ value of 0.9, 5) HS1 with a *R* ^2^ value of 0.5, 6) HS1 with a *R* ^2^ value of 0.1, 7) moderately heteroscedastic data (HS2) with a *R* ^2^ value of 0.9, 8) HS2 with a *R* ^2^ value of 0.5, 9) HS2 with a *R* ^2^ value of 0.1, 10) severely heteroscedastic data (HS3) with a *R* ^2^ value of 0.9, 11) HS3 with a *R* ^2^ value of 0.5, 12) HS3 with a *R* ^2^ value of 0.1.

HS0 is generated using a linear regression formula, which is represented as:

$$Y=0.5+0.5X_{i}+e_{i}\left(for\;observation\;i\right),where\;e_{i}∼N\;\left(0,\sigma_{\varepsilon }^{2}\right).$$

Here, $X_{i}$ is an independent variable for observation *i*, ranging from 1 to 2,000; the intercept and slope coefficients of *X*_*i*_ are both 0.5. The residual term, *e*_*i*_, represents the residual of observation *i* on dependent variables such as *Y*, and follows a normal distribution with a mean of 0 and variance of *σ*_*ε*_^*2*^. The value of *σ*_*ε*_^*2*^ represents the variance of the random error term (*e*_*i*_) for the *i*th observation. The variability of the errors is constant for different values of *X*_*i*_. Consequently, *Y* is dependent on observation *i* and is calculated using the equation 0.5 + 0.5*X*_*i*_ + *e*_*i*_. In addition, for HS0, each *R* ^2^ value of 0.9, 0.5, and 0.1 satisfies the following equation:

$$Y=0.5+0.5X_{i}+e_{i}\left(for\;observation\;i\right),where\;e_{i}∼N\left(0,55^{2}\right);$$

$$Y=0.5+0.5X_{i}+e_{i}\left(for\;observation\;i\right),where\;e_{i}∼N\left(0,285^{2}\right);$$

$$Y=0.5+0.5X_{i}+e_{i}\left(for\;observation\;i\right),where\;e_{i}∼N\left(0,800^{2}\right).$$

HS1 is generated using a linear regression formula, which is represented as:

$$Y=0.5+0.5X_{i}+e_{i}\left(for\;observation\;i\right),where\;e_{i}∼N\left(0,\sigma_{\varepsilon i}^{2}\right).$$

Here, $X_{i}$ is independent variable for observation *i*, ranging from 1 to 2,000; the intercept and slope coefficients of *X*_*i*_ are both 0.5. The residual term, *e*_*i*_, represents the residual of observation *i* on dependent variables such as *Y*, and follows a normal distribution with a mean of 0 and variance of *σ*_*εi*_^*2*^. The value of *σ*_*ε*_^*2*^ represents the variance of the random error term (*e*_*i*_) for the *i*th observation. The variance of the error term (*e*_*i*_) for the *i*th observation, denoted by *σ*_*εi*_^*2*^, indicates that the variability of the errors differs for various values of *X*_*i*_. For HS1, each *R* ^2^ value of 0.9, 0.5, and 0.1 satisfies the following equation:

$$Y=0.5+0.5X_{i}+e_{i}\left(for\;observation\;i\right),where\;e_{i}∼N\left(0,{\left(0.04X_{i}+60\right)}^{2}\right);$$

$$Y=0.5+0.5X_{i}+e_{i}\left(for\;observation\;i\right),where\;e_{i}∼N\left(0,{\left(0.09X_{i}+190\right)}^{2}\right);$$

$$Y=0.5+0.5X_{i}+e_{i}\;\left(for\;observation\;i\right),where\;e_{i}∼N\left(0,{\left(0.3X_{i}+500\right)}^{2}\right).$$

HS2 is generated using a linear regression formula, which is represented as:

$$Y=0.5+0.5X_{i}+e_{i}\left(for\;observation\;i\right),where\;e_{i}\;∼\;N\left(0,\sigma_{\varepsilon i}^{2}\right).$$

Here, $X_{i}$ is the independent variable for observation *i*, ranging from 1 to 2,000; the intercept and slope coefficients of *X*_*i*_ are both 0.5. The residual term, *e*_*i*_, represents the residual of observation *i* on dependent variables such as *Y*, and follows a normal distribution with a mean of 0 and variance of *σ*_*εi*_^*2*^. The value of *σ*_*ε*_^*2*^ represents the variance of the random error term (*e*_*i*_) for the *i*th observation. The variance of the error term (*e*_*i*_) for the *i*th observation, denoted by *σ*_*εi*_^*2*^, indicates that the variability of the errors differs for various values of *X*_*i*_. For HS2, each *R* ^2^ value of 0.9, 0.5, and 0.1 satisfies the following equation:

$$Y=0.5+0.5X_{i}+e_{i}\left(for\;observation\;i\right),where\;e_{i}∼N\left(0,{\left(0.05X_{i}+40\right)}^{2}\right);$$

$$Y=0.5+0.5X_{i}+e_{i}\left(for\;observation\;i\right),where\;e_{i}∼N\left(0,{\left(0.15X_{i}+130\right)}^{2}\right);$$

$$Y=0.5+0.5X_{i}+e_{i}\;\left(for\;observation\;i\right),where\;e_{i}∼N\left(0,{\left(0.5X_{i}+300\right)}^{2}\right).$$

HS3 is generated using a linear regression formula, which is represented as:

$$Y=0.5+0.5X_{i}+e_{i}\left(for\;observation\;i\right),where\;e_{i}∼N\left(0,\sigma_{\varepsilon }^{2}\right).$$

Here, $X_{i}$ is the independent variable for observation *i*, ranging from 1 to 2,000; the intercept and slope coefficients of *X*_*i*_ are both 0.5. The residual term, *e*_*i*_, represents the residual of observation *i* on dependent variables such as *Y*, and follows a normal distribution with a mean of 0 and variance of *σ*_*εi*_^*2*^. The value of *σ*_*ε*_^*2*^ represents the variance of the random error term (*e*_*i*_) for the *i*th observation. The variance of the error term (*e*_*i*_) for the *i*th observation, denoted by *σ*_*εi*_^*2*^, indicates that the variability of the errors differs for various values of *X*_*i*_. For HS3, each *R* ^2^ value of 0.9, 0.5, and 0.1 satisfies the following equation:

$$Y=0.5+0.5X_{i}+e_{i}\left(for\;observation\;i\right),where\;e_{i}∼N\left(0,{\left(0.08X_{i}\right)}^{2}\right);$$

$$Y=0.5+0.5X_{i}+e_{i}\left(for\;observation\;i\right),where\;e_{i}∼N\left(0,{\left(0.25X_{i}\right)}^{2}\right);$$

$$Y=0.5+0.5X_{i}+e_{i}\left(for\;observation\;i\right),where\;e_{i}∼N\left(0,{\left(0.8X_{i}\right)}^{2}\right).$$

All simulation processes were performed using R stats packages version 4.0.5 (www.r-project.org). In addition, linear regression (the LM function in R stats package version 4.0.5) was used to estimate the effect size and *R* ^2^ between X and Y.

### Study population and design

We used the UK Biobank resource, a large-scale population-based dataset that recruited over 487,409 individuals aged 40–69 years during 2006–2010 (Collins, 2012). Quality control of the samples was performed using the following filter parameters from the Neale lab (http://github.com/Nealelab/UK_Biobank_GWAS): principal component (PC) analysis calculation filter for selecting unrelated samples; sex chromosome filter for removing aneuploidy; filtering of PCs for European sample selection to determine British ancestry; and filters for selecting self-reported “white-British,” “Irish,” and “white.” The total number of unrelated white British participants amounted to 364,761.

Among the 364,761 unrelated white British samples, we extracted 10,000 samples for the calculation of the linkage disequilibrium (LD) matrix. The remaining 354,761 samples were divided into two subsets: the GWAS set (*N* = 177,380) and the PRS set (*N* = 177,381). The GWAS set was used for GWAS and consisted of unrelated white British Europeans (*N* = 177,380) and their phenotypic information was collected during the initial assessment period (2006–2010; instance = 0). The individual PRS for 15 traits was estimated using LDpred2 in the PRS set (*N* = 177,381), whose phenotypic information was also collected during the initial assessment period (2006–2010; instance = 0). Next, we randomly selected 80% of the individuals in the PRS set as the modeling set (*N* = 141,905), in which linear regression models between PRS and traits were generated and evaluated for heteroscedasticity. The remaining 20% of individuals in the PRS set (*N* = 35,476) were designated as the validation set, which was used to investigate the prediction accuracy of the PRS model (Supplementary Figure S1).

For the replication analysis, we extracted the unrelated white British samples from the UK Biobank resource, which satisfied the following data filed criteria: 1) coded as “Yes” in the UK Biobank PRS release testing subgroup (field ID: 26200); 2) coded as “No kinship found” in the “genetic kinship to other participants” field; and 3) identified as having a white British, Irish, or White background (field ID: 21000). Lastly, we excluded the 364,761 samples used in the initial analysis, and the remaining 23,620 unrelated white British samples were used for the replication analysis.

### Ethics approval and consent to participate

All participants provided signed consent to participate in the UK Biobank (Biobank, 2007). The UK Biobank was granted ethical approval to collect participant data by the North West Multicenter Research Ethics Committee, covering the United Kingdom; the National Information Governance Board for Health and Social Care, covering England and Wales; and the Community Health Index Advisory Group, covering Scotland. The UK Biobank possesses generic Research Tissue Bank approval granted by the National Research Ethics Service (http://www.hra.nhs.uk/). This allows applicants to conduct research on UK Biobank data without obtaining separate ethical approvals. Access to the UK Biobank data was granted under application no. 83990: “Genetic and environmental analysis for disease prediction models.”

### Phenotype data

Fifteen quantitative traits were selected based on 1) over 5% heritability of traits (Pulit et al., 2019; Watanabe et al., 2020), 2) over 250,000 sample size in unrelated white-British European samples (https://biobank.ndph.ox.ac.uk/showcase/). The following 15 quantitative traits were selected: alanine aminotransferase (ALT) (field ID:30620), alkaline phosphatase (ALP) (field ID:30610), aspartate aminotransferase (AST) (field ID:30650), body mass index (BMI) (field ID:21001), cholesterol (field ID:30690), creatinine (field ID:30700), cystatin C (field ID:30720), forced expiratory volume in one second (FEV1), and forced vital capacity (FVC) ratio (FFR) (field ID:20258), height (field ID:50), phosphate (field ID:30810), platelet count (field ID:30080), red blood cell count (RBC) (field ID:30010), total protein (TP) (field ID:30860), triglycerides (TG) (field ID:30870), and waist-to-hip ratio adjusted for BMI (WHR_adjBMI_) calculated by waist circumference (field ID:48), hip circumference (field ID:49), and BMI (Pulit et al., 2019). Among the 15 quantitative traits, the FFR value is the only value in which a decreasing value indicates the occurrence of lung-related disease.

Individuals known to be on lipid-lowering medications (Field ID: 6153, 6177) were excluded from the TG and cholesterol analyses (Willer et al., 2013) to minimize the treatment bias.

To prevent the bias of prediction accuracy from extreme trait outliers, we excluded the samples showing extreme trait outlier satisfaction (Iida et al., 2020): Y < Q1 − 3IQR or Y > Q3 + 3IQR [Q1: 25th percentile, Q3: 75th percentile, IQR: interquartile range (Q3 − Q1)] (Supplementary Figure S1).

### Genotype data

The 487,409 UK Biobank (UKB) participants were genotyped using the UKB Axiom Array and the United Kingdom BiLEVE Axiom Array from Affymetrix (Sudlow et al., 2015; Bycroft et al., 2018). Genotypes were imputed using the Haplotype Reference Consortium (HRC) and UK10K haplotype resource (McCarthy et al., 2016). Next, we performed quality control of SNPs using PLINK v.1.90 (Purcell et al., 2007) based on the following exclusion criteria: SNPs with missing genotype call rates >0.05, minor allele frequency <0.01, Hardy-Weinberg equilibrium *p*-value < 1.00 *×* 10^−6^, insertion-deletion 780000. Finally, 1,149,057 SNPs were extracted for further analyses after referring to HapMap 3 SNPs and strand-ambiguous SNPs (that is, SNPs with the allele A/T or C/G) (International HapMap Consortium, 2003; International HapMap Consortium, 2005; Agrawal et al., 2022).

### Estimation of genome-wide polygenic risk score

We estimated the PRS using LDpred2 version 1.4.7, a developed algorithm that uses a Bayesian approach to polygenic risk scoring. LDpred2 considers the LD relationship between SNPs and reweights the effect size of SNPs estimated by GWAS (Prive et al., 2020). First, we calculated the LD correlation matrix among 1,149,057 SNPs (HapMap 3 variant) using 10,000 unrelated white British samples, which was randomly extracted from 364,761 unrelated White-British samples. Second, we reweighted the effect size of SNPs estimated by GWAS (Vilhjalmsson et al., 2015; Prive et al., 2020). Each SNP was assigned a weight based on the LD-adjusted effect size using an infinitesimal model of LDpred2, which assumes that all genetic variants are causal. Finally, we constructed individual PRSs as the sum of the weighted risk effect size of SNPs in the PRS set. The PRS of an individual *j*, as a weighted sum of SNP allele counts (Ni et al., 2021; Maj et al., 2022) was formulated as:

$$\hat{{PRS}_{j}}=\sum_{i=1}^{m}\hat{b_{i}}x_{ij},$$

where *m* is the number of SNPs included, $\hat{b_{i}}$ is the estimated reweight for the effect size of SNP *i*, $x_{ij}$ is the number (0, 1, or 2) of trait-associated alleles of SNP *i* in individual *j*.

As described above, increasing FFR values indicate improved health. Therefore, we observed only a decrease in the FFR values as the PRS values increased. To unify the direction of effect size, we substituted the reference alleles with alternative alleles for the effect allele of SNPs on the FFR value. As a result, all 15 quantitative trait values increased as the PRS values increased.

### Heteroscedasticity test

We used the Breusch-Pagan (BP), Score, and F tests in the ordinary least squares (OLS) R package version 0.5.3 to evaluate the heteroscedasticity of a trait across the PRS (Breusch and Pagan, 1979; Cook and Weisberg, 1983). The BP, Score, and F tests are commonly used statistical tests to assess heteroscedasticity in a regression model.

The BP test determines whether the variance of the residuals is constant across all independent variables by assessing the association between the squared residuals and the independent variables. A significant result suggests the presence of heteroscedasticity. The resulting test statistic follows a chi-squared distribution with degrees of freedom equal to the number of independent variables, and the resulting *p*-value is used to determine whether to reject the null hypothesis of homoscedasticity in favor of the alternative hypothesis of heteroscedasticity.

The Score test uses the likelihood ratio test to compare the fit of the original regression model to a modified model that includes an additional term to account for heteroscedasticity. The resulting test statistic follows a chi-squared distribution with degrees of freedom equal to the number of parameters in the additional term, and its significance is interpreted similarly to that of the BP test.

Similarly, the F test assesses the overall model fit and can also be used to test for heteroscedasticity. It compares the fit of the original regression model to a modified model that includes an additional term to account for heteroscedasticity, with the resulting test statistic following an F distribution with degrees of freedom equal to the difference in the number of parameters between the two models.

The amount of heteroscedasticity was quantified by determining the ratio of the mean of the absolute residuals for the largest 10 percent PRS (G10) and the smallest 10 percent PRS (G1).

### Polygenic score (PGS) catalog

The PGS catalog is an extensive online database that gathers and curates published PRSs from various studies (Lambert et al., 2021). We utilized the PGS catalog database to obtain large-scale GWAS summary statistics for 15 quantitative traits, using the following PGS scores: PGS002158 for ALT, PGS002157 for ALP, PGS002159 for AST, PGS002162 for BMI, PGS002108 for cholesterol, PGS002163 for creatinine, PGS002165 for cystatin C, PGS002146 for height, PGS002216 for phosphate, PGS002191 for platelet count, PGS002123 for RBC, PGS002219 for TP, PGS002197 for TG (Prive et al., 2022), PGS001801 for FFR (Moll et al., 2020), and PGS000299 for WHR_adjBMI_ (Xie et al., 2020). The PGS catalog database included the reweighted effect size, reference allele of SNPs, and list of SNPs. We utilized the information to estimate the individual PRSs using PLINK v.1.90.

### Statistical analysis

We performed GWAS on 15 quantitative traits in the GWAS set using the linear regression model by PLINK v.2.00 (Purcell et al., 2007). The following linear regression formula was used:

Trait = *β*_1_ genotype + *β*_2_ age + *β*_3_ sex + *β*_4_ genotyping array + *β*_5_ PC1 + *β*_6_ PC2 + *β*_7_ PC3 + *β*_8_ PC4 + *β*_9_ PC5 + *β*_10_ PC6 + *β*_11_ PC7 + *β*_12_ PC8 + *β*_13_ PC9 + *β*_14_ PC10, where*, β*_1_ denotes the effect size of genotype (coded as 0, 1 or 2), *β*_2_ denotes the effect size of age at recruitment (ranging from 40 to 69), *β*_3_ denotes the effect size of sex (coded as 0 or 1 for female or male, respectively), *β*_4_ denotes the effect size of genotyping array (coded as 0 or 1 for the UKB Axiom Array and the UK BiLEVE Axiom Array) (Sudlow et al., 2015), *β*_5_ *∼ β*_14_ denote the effect size of PC1 ∼ PC10, which accounts for any population stratification or ancestry differences between individuals in the study.

Correlation was tested between heritability and the *R* ^2^ of PRS-based prediction model using the Pearson correlation method in R stats packages version 4.1.0. To investigate the relationship between the X and Y variables in the simulation, as well as between each of the 15 PRS and trait values in both the PRS and replication sets, a linear regression model was performed using the R package version 4.1.0.

We depicted the scatter plot, line plot, histogram, and bar plot using ggplot2 version 3.3.6 in R. Heteroscedasticity for linear regression models was detected using the Breusch-Pagan (BP), score, and F test in ordinary least squares (OLS) R packages version 0.5.3 provided in R (Breusch and Pagan, 1979; Cook and Weisberg, 1983).

In the modeling set (*N* = 141,905), we obtained the effect size (*β*_1_) of the PRS on each trait using linear regression model in R package version 4.1.0. Additionally, we estimated the intercept values (*β*_0_) of the linear regression model between the PRS and trait values. Next, in the validation set (*N* = 35,476), we estimated the predicted trait values using the following formula:

$${Ŷ}_{ij}=\beta_{0}+\beta_{1}{PRS}_{ij}.$$

Here, *Ŷ*_*ij*_ represents the predicted value of trait *j* for in dividual *i*. *β*_0_ is the intercept value that was estimated through linear regression between the PRS and trait *j* in the modeling set. *β*_1_ is the effect size of the PRS that was calculated through linear regression between the PRS and trait *j* in the modeling set. *PRS*_*ij*_ refers to the individual PRS on each trait (*j*).

In addition, we estimated the individual error of PRS models as follows:

$$Error=Y_{ij}-{Ŷ}_{ij}$$

Here, *Ŷ*_*ij*_ represents the predicted value of trait *j* for individual *i* based on the linear regression model using the PRS, *Y*_*ij*_ represents the actual value of trait *j* for the individual *i*.

The error rate outside 1 SD of errors in each decile group of PRS is designated as follows: 1) calculate the individual error of the PRS model, 2) find an error more than ±1 SD error value from the mean of the error value, 3) compute the proportion of errors found in 2) by PRS groups.

## Results

### Understanding heteroscedasticity using simulation data

**FIGURE 1:**
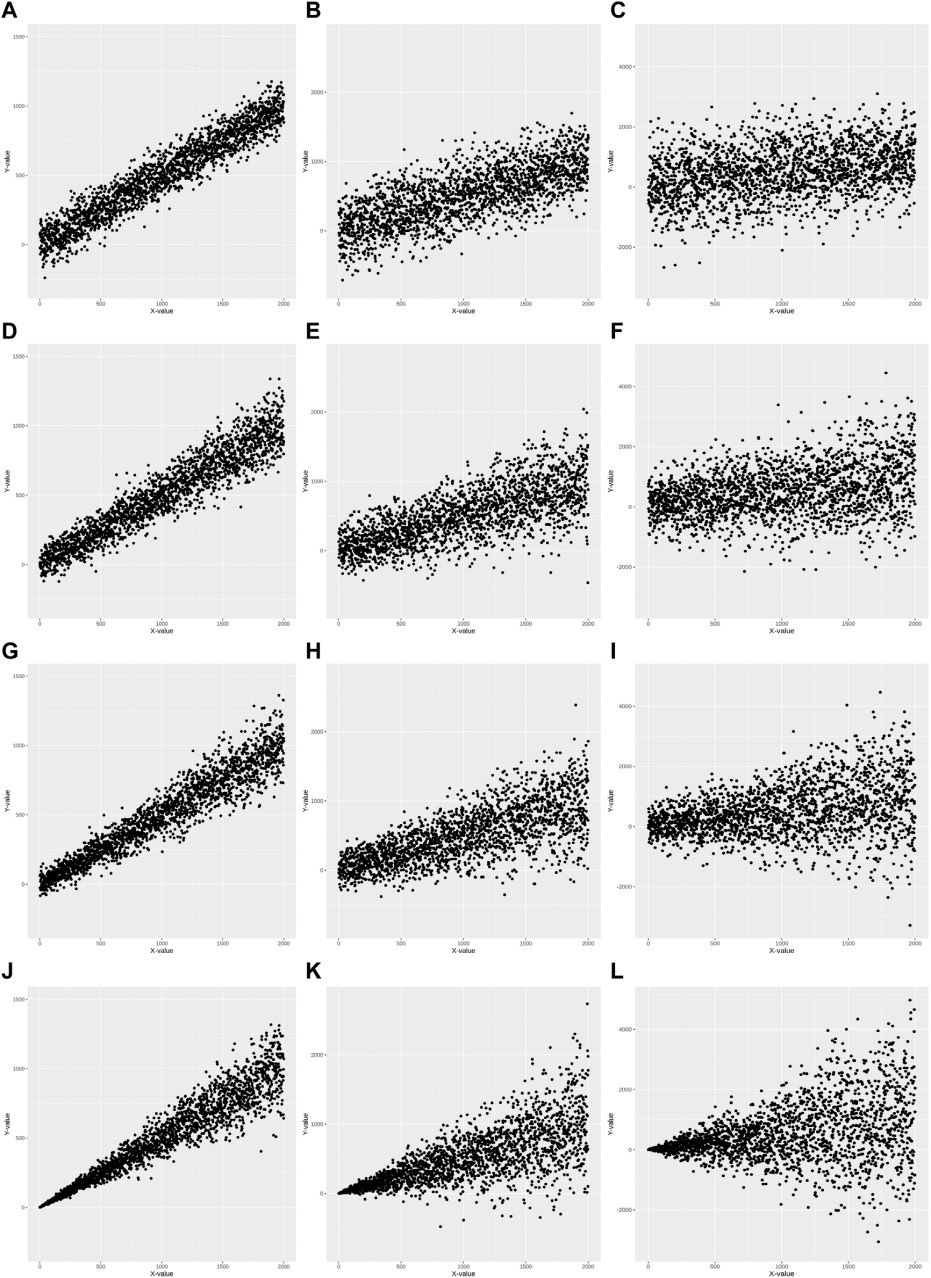
Scatter plots of 12 simulation data. Homoscedasticity (HS0) is **(A–C)**. Mild heteroscedasticity (HS1) is **(D–F)**. Moderate heteroscedasticity (HS2) is **(G–I)**. Severe heteroscedasticity is (HS3) is **(J–L)**. The *R* ^2^ value of **(A, D, G, J)** is 0.9. The *R* ^2^ value of **(B, E, H, K)** is 0.5. The *R* ^2^ value of **(C, F, I, L)** is 0.1.

**FIGURE 2:**
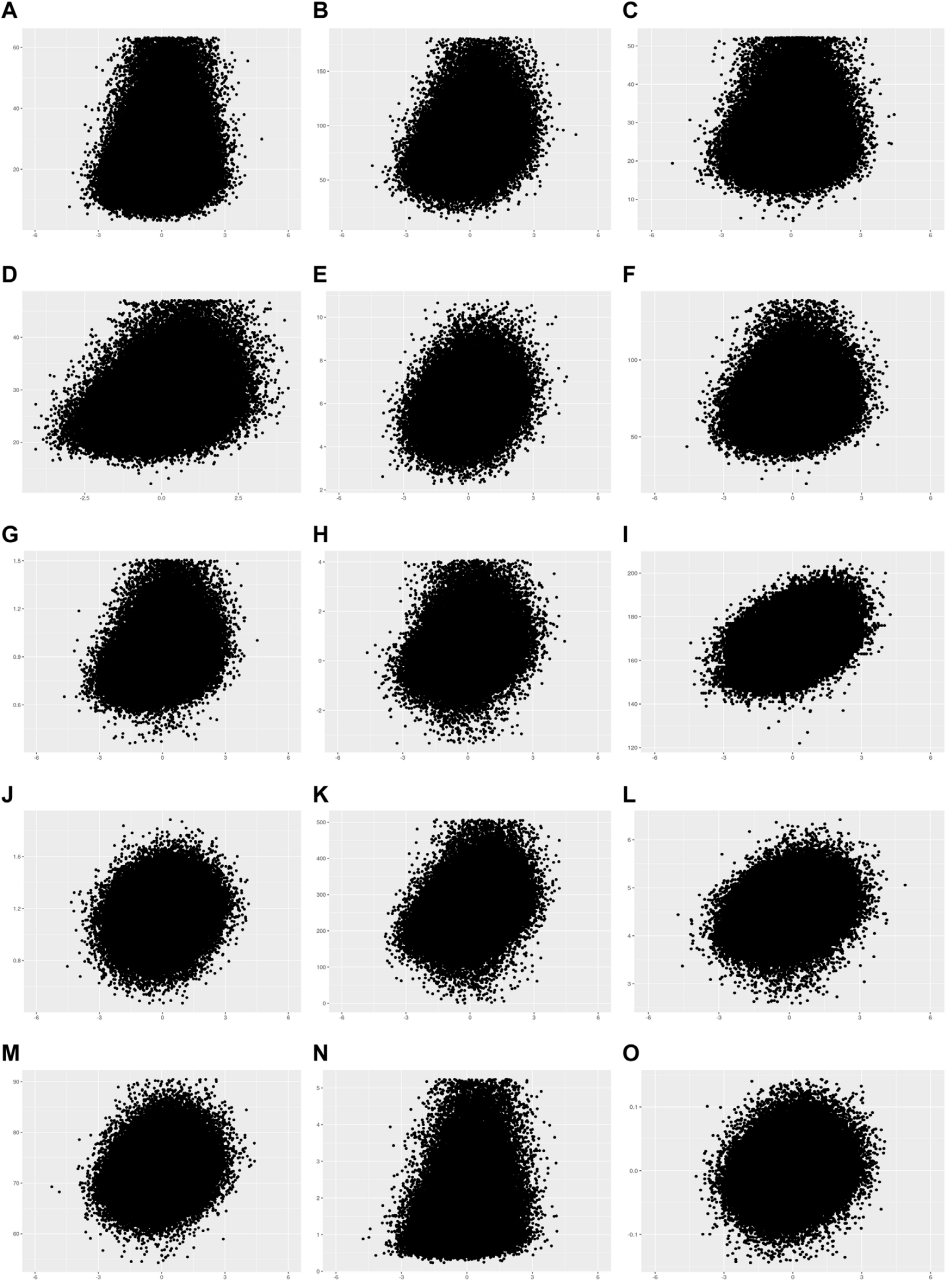
Scatter plots of 15 quantitative traits in the PRS set. The *X*-axis is the standardized PRS. The *Y*-axis is the phenotype value. **(A)** Alanine aminotransferase, **(B)** Alkaline phosphatase, **(C)** Aspartate aminotransferase, **(D)** Body mass index, **(E)** Cholesterol, **(F)** Creatinine, **(G)** Cystatin C, **(H)** FEV1/FVC ratio, **(I)** Height, **(J)** Phosphate, **(K)** Platelet count, **(L)** Red blood cell count, **(M)** Total protein, **(N)** Triglycerides, **(O)** Waist-to-hip ratio adjusted for BMI.

**FIGURE 3:**
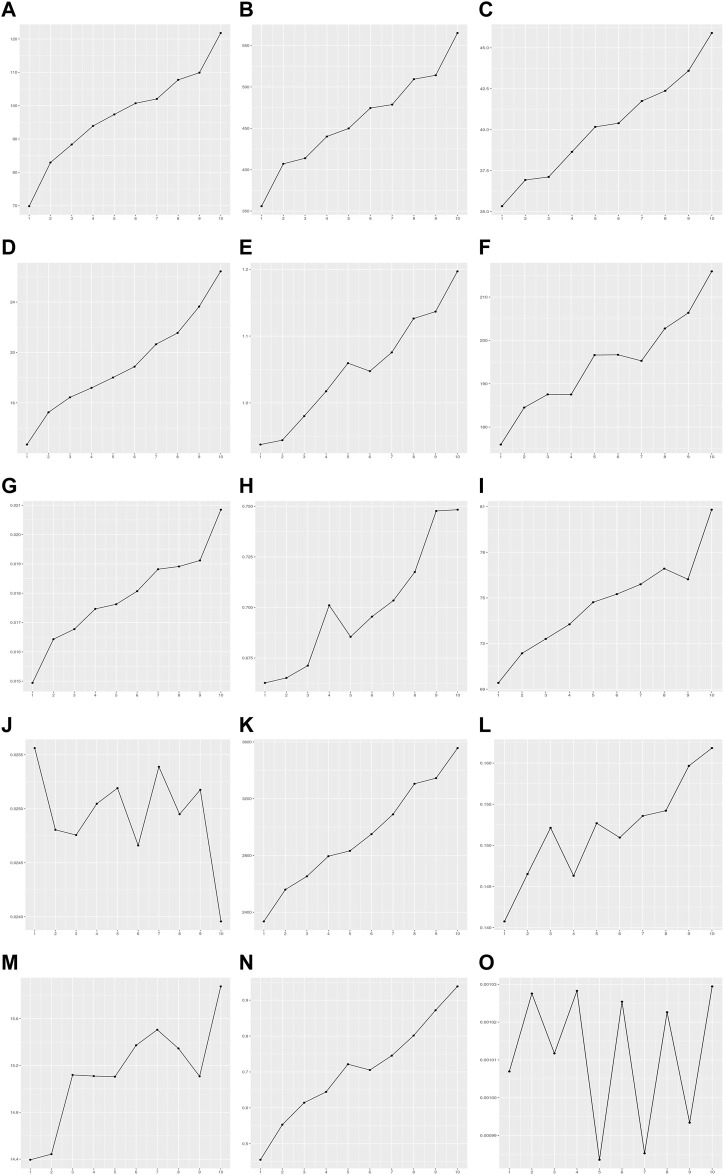
Variance of residuals in the PRS model of 15 quantitative traits. The *X*-axis is the decile group of PRS: G1-G10 from left to right on the *X*-axis. The *Y*-axis is the variance of residuals on each decile group of PRS estimated by the PRS model. **(A)** Alanine aminotransferase, **(B)** Alkaline phosphatase, **(C)** Aspartate aminotransferase, **(D)** Body mass index, **(E)** Cholesterol, **(F)** Creatinine, **(G)** Cystatin C, **(H)** FEV1/FVC ratio, **(I)** Height, **(J)** Phosphate, **(K)** Platelet count, **(L)** Red blood cell count, **(M)** Total protein, **(N)** Triglycerides, **(O)** Waist-to-hip ratio adjusted for BMI.

Heteroscedasticity is defined as a model with unequal variance of residuals for each level of the predictor variable(s) fitted to the linear regression model (Andy, 2009). Simulation data for twelve sets was generated to better understand diverse heteroscedasticities using common simulation conditions: a predictor (independent) variable, X, was statistically associated with the criterion (dependent) variable, Y. Twelve sets were generated under these conditions according to the following scenarios: 1) three simulation datasets for homoscedasticity (HS0) with respective *R* ^2^ values of 0.9, 0.5, and 0.1 (Figures 1A–C); 2) three simulation datasets for mild heteroscedasticity (HS1) with respective *R* ^2^ values of 0.9, 0.5, and 0.1 (Figures 1D–F); 3) three simulation datasets for moderate heteroscedasticity (HS2) with respective *R* ^2^ values of 0.9, 0.5, and 0.1 (Figures 1G–I); and 4) three simulation datasets for severe heteroscedasticity (HS3) with respective *R* ^2^ values 0.9, 0.5, and 0.1 (Figures 1J–L).

Statistical tests of heteroscedasticity and association were performed including constructing linear prediction models and validating their performance for each simulation dataset. Each simulation dataset (HS0, HS1, HS2, and HS3) was randomly divided into two subsets: modeling set (50%) and validation set (50%) to perform all tests using independent datasets. An association analysis using a linear regression model was performed between X and Y in the modeling set (Table 1). For each of the four heteroscedasticity levels (HS0, HS1, HS2, and HS3), three models demonstrated significant positive associations between variables X and Y, with effect sizes ranging from 0.47 to 0.55 and *p*-values < 2.00E-16. The *R* ^2^ values varied between models and heteroscedasticity levels, ranging from 0.10 to 0.91 (Table 1). Formal statistical tests (BP test, score test, and F-test) were performed to determine the presence of heteroscedasticity in each modeling set (Table 2) (Breusch and Pagan, 1979; Cook and Weisberg, 1983). HS0 did not show statistical significance for heteroscedasticity in any of the three tests. However, HS1, HS2, and HS3 were statistically significant for these three heteroscedasticity tests (Table 2). Additionally, no differences were found across all decile groups for HS0 after plotting the variance of residuals for each decile group of X using a line graph (Supplementary Figures S2A–C). However, the graphs for HS1, HS2, and HS3 show that the variance of residuals gradually increased from the 1st group decile of X (G1) to the 10th group decile of X (G10) (Supplementary Figures S2D–L). Heteroscedasticity was quantified using the ratio of the mean absolute residuals between G1 and G10 (Gelfand, 2015). For HS0, the ratio of the mean absolute residual was 0.95 (*R* ^2^ = 0.90), 0.99 (*R* ^2^ = 0.50), and 0.90 (*R* ^2^ = 0.10). For HS1, the ratio of the mean absolute residual was 1.99 (*R* ^2^ = 0.90), 1.83 (*R* ^2^ = 0.50), and 2.29 (*R* ^2^ = 0.10). For HS2, the ratio of the mean absolute residual was 3.10 (*R* ^2^ = 0.90), 3.59 (*R* ^2^ = 0.51), and 3.19 (*R* ^2^ = 0.10). For HS3, the ratio of the mean absolute residual was 17.82 (*R* ^2^ = 0.91), 17.73 (*R* ^2^ = 0.50), and 20.61 (*R* ^2^ = 0.10) (Supplementary Table S1; Supplementary Figure S3). The ratio of the mean absolute residual between G1 and G10 is close to 1 under the assumption of homoscedasticity. However, the ratio of the mean absolute residual was greater than 1 under the assumption of heteroscedasticity, and this value gradually increased as heteroscedasticity changed from mild to severe.

**TABLE 1:** Association results of 12 simulation data.

Dataset	Effect size^a^	Standard error	*p*-value	*R* ^2^
HS0 (Homoscedasticity)	0.50	0.01	<2.00E-16	0.90
	0.49	0.02	<2.00E-16	0.50
	0.47	0.04	<2.00E-16	0.10
HS1 (Heteroscedasticity)	0.51	0.01	<2.00E-16	0.90
	0.49	0.02	<2.00E-16	0.50
	0.49	0.05	<2.00E-16	0.10
HS2 (Heteroscedasticity)	0.50	0.01	<2.00E-16	0.90
	0.51	0.02	<2.00E-16	0.51
	0.49	0.05	<2.00E-16	0.10
HS3 (Heteroscedasticity)	0.50	0.01	<2.00E-16	0.91
	0.50	0.02	<2.00E-16	0.50
	0.55	0.05	<2.00E-16	0.10

^a^ Magnitude of the relationship between X-values and Y-values.

**TABLE 2:** Heteroscedasticity tests of 12 simulation data.

Dataset	*R* ^2^	Statistical index	Breusch-Pagan	Score	F
HS0 (Homoscedasticity)	0.90	*χ2*	0.21	0.23	0.23
		*p*-value	6.46E-01	6.29E-01	6.29E-01
	0.50	*χ2*	0.01	0.01	0.01
		*p*-value	9.06E-01	9.03E-01	9.03E-01
	0.10	*χ2*	3.86	3.73	3.74
		*p*-value	4.94E-02	5.35E-02	5.35E-02
HS1 (Heteroscedasticity)	0.90	*χ2*	74.60	63.36	67.51
		*p*-value	5.76E-18	1.72E-15	6.47E-16
	0.50	*χ2*	89.53	71.82	77.22
		*p*-value	3.02E-21	2.36E-17	6.56E-18
	0.10	*χ2*	84.39	59.91	63.60
		*p*-value	4.06E-20	9.94E-15	4.16E-15
HS2 (Heteroscedasticity)	0.90	*χ2*	170.76	102.92	114.5
		*p*-value	5.06E-39	3.48E-24	2.26E-25
	0.51	*χ2*	187.78	118.99	134.79
		*p*-value	9.72E-43	1.05E-27	2.55E-29
	0.10	*χ2*	213.25	150.63	176.99
		*p*-value	2.68E-48	1.26E-34	2.69E-37
HS3 (Heteroscedasticity)	0.91	*χ2*	356.08	190.89	235.46
		*p*-value	2.01E-79	2.03E-43	7.17E-48
	0.50	*χ2*	344.52	161.59	192.34
		*p*-value	6.61E-77	5.09E-37	3.99E-40
	0.10	*χ2*	381.92	196.67	244.33
		*p*-value	4.76E-85	1.11E-44	1.97E-49

*χ2*: chi-square value.

A consequence of heteroscedasticity in regression analysis is that coefficient estimates remain unbiased and consistent, but are no longer efficient or less accurate (Andy, 2009). This is because the regression model weighs equally for all samples, such as the values of X in our simulation data, even though the difference in variance of the residuals may occur. Therefore, we investigated whether heteroscedasticity affects the prediction accuracy at each level of the predictor variable(s) (or group) fitted to the model. We predicted the values of Y by applying the regression model generated for each modeling set using the validation set. The error in each sample by the prediction model was estimated as follows: error = *Y*_*i*_ - Ŷ_i_ *.* Also the error rate was defined as the ratio of samples outside 1 standard deviation (SD) of error values. The Supplementary Table S2 summarized the range of error rates calculated for each decile group of X, providing an indicator of prediction accuracy (Supplementary Figure S4). This indicates that the error rate was similar in each group under homoscedasticity, but tended to increase according to the decile group under heteroscedasticity.

### Basic characteristics of 15 quantitative traits

We applied these analysis schemes from the simulation results to real data from the UK Biobank to test for heteroscedasticity between the PRSs and traits (Supplementary Figure S1). The basic characteristics of the 15 quantitative traits in each dataset (GWAS and PRS sets) are summarized in Supplementary Table S3.

We selected 15 quantitative traits based on heritability (*h* ^*2*^) explained by common variants (Pulit et al., 2019; Watanabe et al., 2019) and performed GWAS for 15 quantitative traits in the GWAS set using linear regression analysis adjusted for age, sex, array, and PC1 ∼ 10. We estimated the *h* ^*2*^ and intercept representing the genomic inflation using linkage disequilibrium score regression (LDSC) based on GWAS summaries for 15 quantitative traits (Supplementary Table S4) (Vilhjalmsson et al., 2015). The highest *h* ^*2*^ was observed in the height (0.43), and the lowest *h* ^*2*^ was observed in aspartate aminotransferase (AST) (0.06).

Next, we calculated PRSs of individuals included in the PRS set. The effect sizes of each genetic variant were reweighted based on its effect size and statistical significance from 15 GWAS summary statistics using LDpred2 (Prive et al., 2020). The PRSs of individuals were calculated using these reweighted summary statistics. All the PRSs were normally distributed (Supplementary Figure S5) and the value of each trait increased as standardized PRS increased (Figure 2; Supplementary Figure S6). The results of the association analysis between each PRS and trait indicated that the effect sizes (or beta coefficients) of the PRSs was between 0.01 and 19.52, and the *R* ^*2*^ representing the goodness-of-fit measure for the regression model was between 0.02 and 0.13 (Supplementary Table S5). Height showed the highest *R* ^2^ value, and ALT and AST showed the lowest *R* ^2^ value (Supplementary Table S5). The *R* ^2^ value was statistically correlated with heritability (Pearson’s correlation coefficient *r* = 0.87, and *p*-value = 2.31E-05).

### Identification of heteroscedasticity and its influence on prediction accuracy using UK Biobank data

The BP test, score test, and F tests were performed using the modeling set to detect the heteroscedasticity of PRS models in 15 quantitative traits. Thirteen out of fifteen PRS models exhibited significant heteroscedasticity based on the Bonferroni corrected *P*-threshold of 3.33E-03 (= 0.05/15 quantitative traits) (Table 3). However, heteroscedasticity was not confirmed in the two PRS models for phosphate and waist-to-hip ratio adjusted for BMI (WHR_adjBMI_). The variance of residuals for each decile group of PRS from G1 to G10 showed that the variances of residuals in these 13 PRS models tended to increase as PRS increased (Figure 3). Heteroscedasticity was quantified using the ratio of mean absolute residuals between G1 and G10 (Gelfand, 2015): 1.53 for triglycerides (TG), 1.47 for BMI, 1.38 for alanine aminotransferase (ALT), 1.30 for alkaline phosphatase (ALP), 1.24 for platelet count, 1.21 for cystatin C, 1.18 for AST, 1.14 for cholesterol, 1.11 for creatinine, 1.09 for forced expiratory volume in one second and forced vital capacity ratio (FFR), 1.07 for red blood cell count (RBC), 1.07 for height, and 1.06 for total protein (TP) (Supplementary Figure S7; Supplementary Table S6).

**TABLE 3:** Heteroscedasticity tests of 15 quantitative traits in the modeling set.

Field ID	Trait	Statistical index	Breusch-Pagan	Score	F
30620	Alanine aminotransferase	*χ2*	1370.68	711.99	715.81
		*p*-value	4.94E-300	7.40E-157	2.85E-157
30610	Alkaline phosphatase	*χ2*	1072.93	696.53	700.14
		*p*-value	2.53E-235	1.70E-153	6.93E-154
30650	Aspartate aminotransferase	*χ2*	398.63	216.88	217.24
		*p*-value	1.09E-88	4.33E-49	3.97E-49
21001	Body mass index	*χ2*	2950.86	1909.06	1935.14
		*p-*value	<1.00E-300	<1.00E-300	<1.00E-300
30690	Cholesterol	*χ2*	273.76	228.77	229.34
		*p-*value	1.72E-61	1.10E-51	9.58E-52
30700	Creatinine	*χ2*	208.71	156.24	156.42
		*p-*value	2.63E-47	7.51E-36	7.19E-36
30720	Cystatin C	*χ2*	543.84	336.74	337.58
		*p-*value	2.76E-120	3.27E-75	2.65E-75
20258	FEV1/FVC ratio	*χ2*	98.11	59.03	59.06
		*p-*value	3.97E-23	1.55E-14	1.54E-14
50	Height	*χ2*	115.76	165.80	165.99
		*p-*value	5.36E-27	6.13E-38	5.85E-38
30810	Phosphate	*χ2*	6.80	6.26	6.26
		*p-*value	9.13E-03	1.23E-02	1.23E-02
30080	Platelet count	*χ2*	1084.69	713.67	717.38
		*p-*value	7.01E-238	3.19E-157	1.26E-157
30010	Red blood cell count	*χ2*	96.34	87.43	87.48
		*p-*value	9.69E-23	8.74E-21	8.62E-21
30860	Total protein	*χ2*	37.21	30.18	30.19
		*p-*value	1.06E-09	3.93E-08	3.93E-08
30870	Triglycerides	*χ2*	1762.06	936.01	945.75
		*p-*value	<1.00E-300	1.46E-205	1.29E-206
Preprocessing	Waist-to-hip ratio adjusted for BMI	*χ2*	0.05	0.04	0.04
		*p-*value	8.20E-01	8.40E-01	8.40E-01

*χ2*: chi-square value, BMI, body mass index; FEV1, Forced expiratory volume in one second, FVC, Forced vital capacity.

**FIGURE 4:**
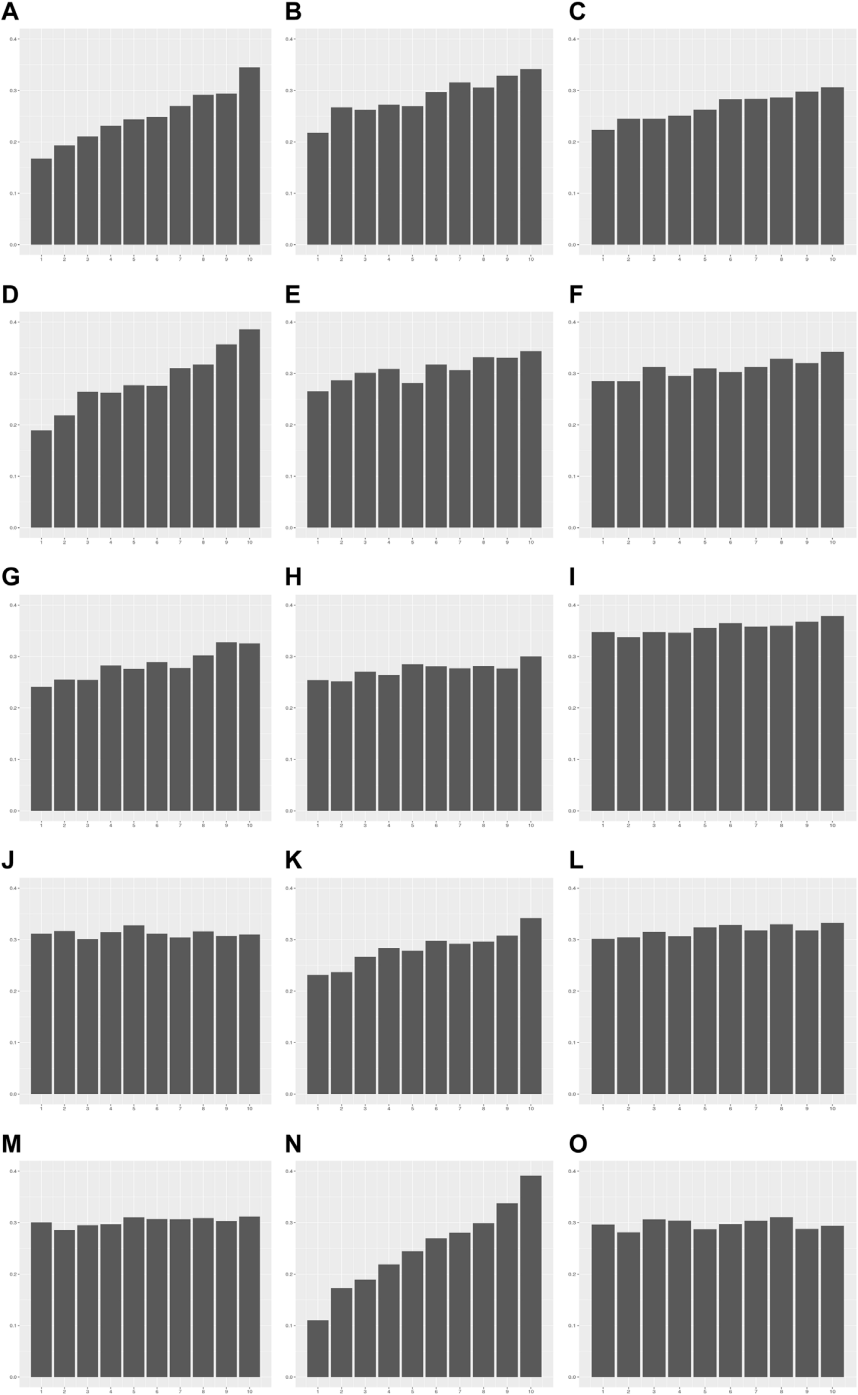
The error rate in each decile group of PRS for 15 quantitative traits. The *X*-axis is the decile group of PRS: G1-G10 from left to right on the *X*-axis. The *Y*-axis is the error rate of the PRS model for each decile group. **(A)** Alanine aminotransferase, **(B)** Alkaline phosphatase, **(C)** Aspartate aminotransferase, **(D)** Body mass index, **(E)** Cholesterol, **(F)** Creatinine, **(G)** Cystatin C, **(H)** FEV1/FVC ratio, **(I)** Height, **(J)** Phosphate, **(K)** Platelet count, **(L)** Red blood cell count, **(M)** Total protein, **(N)** Triglycerides, **(O)** Waist-to-hip ratio adjusted for BMI.

We investigated whether heteroscedasticity affects prediction accuracy at each level of PRS. PRS models developed from the modeling set were applied to the validation set, and the values of each individual trait were predicted (Supplementary Figure S1). The error in each sample by the prediction model was estimated as follows: error = *Y*_*ij*_ - *Ŷ*_*ij*_ (*Materials and methods*). The error rate was defined as the ratio of samples outside 1 SD of error values. The error was calculated in each decile group of the PRS as an indicator of prediction accuracy. The error rate tended to increase as PRS increased from G1 to G10 for the 13 traits showing statistical significance for heteroscedasticity (Figure 4; Supplementary Table S7). For example, the error rates of G1 and G10 in TG were 0.11 and 0.39, respectively. This indicates that the PRS model for TG is 3.55 times more precise in the low PRS group than in the high PRS group (Supplementary Figure S8). These results were similar to the ratios of the mean absolute residuals between G1 and G10 (Supplementary Table S6; Supplementary Figure S7).

### Replication analysis

We conducted a replication analysis to verify the presence of heteroscedasticity in the quantitative traits using a large-scale GWASs in the replication set, independent samples composed of 23,620 European samples from the UK Biobank (*Materials and methods*). We calculated the PRSs of individuals using PGS catalog data base, which resulted in an average *R* ^2^ increase of 0.06–0.13 across 15 quantitative traits in the modeling of the replication set (Supplementary Tables S5, S8). Among the 15 quantitative traits, 10 quantitative traits (ALT, ALP, AST, BMI, cholesterol, creatinine, cystatin C, platelet count, RBC, and TG) showed significant heteroscedasticity in both the prior and replication analyses (*p*-value threshold: 3.33E-03) (Supplementary Table S9).

We quantified heteroscedasticity using the ratio of mean absolute residuals between each decile group of the PRS from G1 to G10 and found that the variance tended to increase as the PRS increased for these 10 PRS models: 1.60 for TG, 1.42 for ALT, 1.34 for BMI, 1.32 for cholesterol, 1.23 for platelet count, 1.22 for ALP, 1.20 for AST, 1.16 for cystatin C, 1.09 for creatinine, and 1.07 for RBC (Supplementary Table S10).

We also investigated whether heteroscedasticity affects prediction accuracy at each level of the PRS. PRS models developed from the modeling of the replication set were applied to the validation of replication set, and the values of each individual trait were predicted (Supplementary Table S11). The error in each sample by the prediction model was estimated as follows: error = *Y*_*ij*_ − *Ŷ*_*ij*_ (*Materials and methods*). The error rate was defined as the ratio of samples outside 1 SD of error values. The error was calculated in each decile group of PRS as an indicator of prediction accuracy. We found that the error rate tended to increase as PRS increased from G1 to G10 for the 10 traits showing statistical significance for heteroscedasticity. For example, the error rates of G1 to G10 in TG were 0.14 and 0.44, respectively, indicating that the PRS model for TG was 3.14 times more precise in the low PRS group than in the high PRS group (Supplementary Table S11). These results were consistent with the ratios of the mean absolute residuals between G1 and G10.

## Discussion

This study estimated the existence of heteroscedasticity between PRSs and 15 quantitative traits using 354,761 Europeans from the UK Biobank. In addition, to validate the presence of heteroscedasticity using an improved PRS performance, we calculated the additional PRSs from the PGS catalog database in 23,620 Europeans from the UK Biobank and evaluated them for heteroscedasticity. As a result, ten out of fifteen quantitative traits showed statistically significant heteroscedasticity between the PRS and each trait. In addition, the prediction accuracy at each level of the of the PRS tended to decrease as the variance of the residuals increased under the condition of heteroscedasticity.

The linear regression model assumes that the variance of the residual is constant for predictor variables (Hayes and Cai, 2007). Most previous studies developed a PRS-based prediction model using a linear regression model and evaluated the predictive performance of the model using the *R* ^*2*^ value (Khera et al., 2019; Ruan et al., 2022; Tanigawa et al., 2022). However, we found that the prediction accuracy (*R* ^*2*^) was different for each level of the PRS in the PRS models with heteroscedasticity. These differences in the prediction performance were caused by the disparity in variance of the residuals according to the PRS (Figure 3). Recently, PRS-based prediction models were constructed and assessed for various traits (Khera et al., 2018; Khera et al., 2019; Tanigawa et al., 2022). However, few studies have examined heteroscedasticity of the PRS model (Sulc et al., 2020; Baek et al., 2022). This study developed a PRS-based prediction model for 15 quantitative traits and investigated whether heteroscedasticity commonly exists in the PRS model. Ten out of fifteen (67%) PRS models showed heteroscedasticity based on three conventional statistical methods: BP, score, and F test. This suggests that PRS models of quantitative traits frequently have heteroscedasticity. Particularly, the variance of residuals increased with higher PRS in the PRS models (Figure 3). For example, the average absolute residuals for the top 10% of PRS in TG was 1.53 times higher than that of the bottom 10% of PRS (Supplementary Table S6).

A key public health need is to identify individuals at high risk of the disease to enable enhanced disease screening or preventive therapies (Khera et al., 2018). Therefore, it is important to assess the performance of the PRS-based prediction model for use as a clinical indicator. The *R* ^2^ value is commonly used as a predictive performance indicator for PRS-based prediction models (Vilhjalmsson et al., 2015; Khera et al., 2019; Prive et al., 2020; Ruan et al., 2022; Tanigawa et al., 2022). However, even though two prediction models showed similar *R* ^2^ values, the prediction accuracies may differ according to the distribution for the variance of the residuals across the predictor variable in each model. Our simulation results showed that different error rates were found in the 12 simulation models with the same *R* ^2^ values but the different distributions for the variance of the residuals (Supplementary Table S2). The error rates of the homoscedastic model (HS0 with an *R* ^2^ of 0.10) were similar across all levels of the predictor variable X, ranging from 23% to 39%. Meanwhile, the error rates of the heteroscedastic models (HS1, HS2, and HS3 with *R* ^2^ values of 0.10) differed according to the predictor variable X, and the difference of the error rates among the three heteroscedastic models depended on the severity of heteroscedasticity; ranges of the error rate were 25%–36% in HS1 with an *R* ^2^ value of 0.10, 1%–48% in HS2 with an *R* ^2^ value of 0.10, and 0%–53% in HS3 with an *R* ^2^ value of 0.10. The error rates in the HS3 model with a *R* ^2^ value of 0.10 indicate that all individuals with G1 group (the lowest PRS decile) were precisely predicted, whereas more than half of the individuals with G10 group (the highest PRS decile) were incorrectly predicted. These results were confirmed in the PRS-based prediction models using the real data from the UK Biobank. The prediction models for phosphate, creatinine and ALT had similar prediction performance (*R* ^2^) as follows; 0.03 for phosphate, 0.03 for creatinine, and 0.02 for ALT (Supplementary Table S5). However, heteroscedasticity was significant only in the creatinine and ALT models, and the ALT model showed more severe heteroscedasticity than that of creatinine (Supplementary Table S6). The ranges of the error rate were 30%–33% in phosphate, 29%–34% in creatinine, and 17%–35% in ALT (Supplementary Table S7) and the ratios of error rates between the G1 and G10 group were 0.99, 1.20, and 2.07 for phosphate, creatinine and ALT, respectively (Supplementary Figure S8). As mentioned, we estimated the level of heteroscedasticity with the ratio of mean absolute residuals for the G1 and G10. These two indicators of error rates and level of heteroscedasticity were highly correlated with each other (Pearson’s correlation coefficient, *r* = 0.89), suggesting the necessity to consider heteroscedasticity to accurately assess the performance of the PRS-based prediction model.

Recently, PRSs were used as predictive biomarkers to identify high-risk disease groups (Khera et al., 2018; Konuma and Okada, 2021; Riveros-Mckay et al., 2021). Khera et al. (2019) found that 83% of the high-risk group of PRS_BMI_ were obese and overweight, while the remaining 17% had a normal BMI range or were underweight. Chen et al. (2021) constructed PRS for ALT, PRS_ALT_ and found that the disease risks (or ORs) between the groups of the bottom 10% and the top 10% of PRS_ALT_ were 1.88 for cirrhosis, and 1.67 for hepatic steatosis. However, differences in prediction accuracy according to PRS groups may occur if heteroscedasticity occurs in PRS models because of differences in residuals for individuals. Accordingly, our results showed that traits exhibiting heteroscedasticity in the PRS model have a larger variance of residuals in the genetically high-risk group based on PRS; this consequently leads to a higher error value. Therefore, it is necessary to test the heteroscedasticity of PRS models to utilize the PRS for the stratification of high-risk disease groups.

Our study had several limitations. First, heteroscedasticity was only analyzed using European participants. Therefore, heteroscedasticity should be evaluated across various ethnic groups. Second, we focused on detecting and understanding heteroscedasticity in the PRS-based prediction models, but did not investigate the causes of heteroscedasticity. It is important to find factors causing the heteroscedasticity in order to improve prediction models more precise.

In conclusion, we identified 10 quantitative traits out of 15 that showed statistical significance for heteroscedasticity using BP, score, and F-test. In these 10 PRS models, we observed that the variances of residuals differed among the PRS groups, and the error rates tended to increase with increasing PRS. This indicates that the accuracy of the predictive model may differ according to PRS values. Therefore, prediction models using PRS for such quantitative traits should consider heteroscedasticity.

## Data Availability

The datasets presented in this study can be found in online repositories. The names of the repository/repositories and accession number(s) can be found in the article/Supplementary Material.
