# Supplementary material for: Investigation of heteroscedasticity in polygenic risk scores across 15 quantitative traits
Source: Front Genet. 2023 May 9;14:1150889. doi: 10.3389/fgene.2023.1150889 (PMC10203621; doi:10.3389/fgene.2023.1150889)
Supplement: Supplementary file 2 [file Image1.pdf]

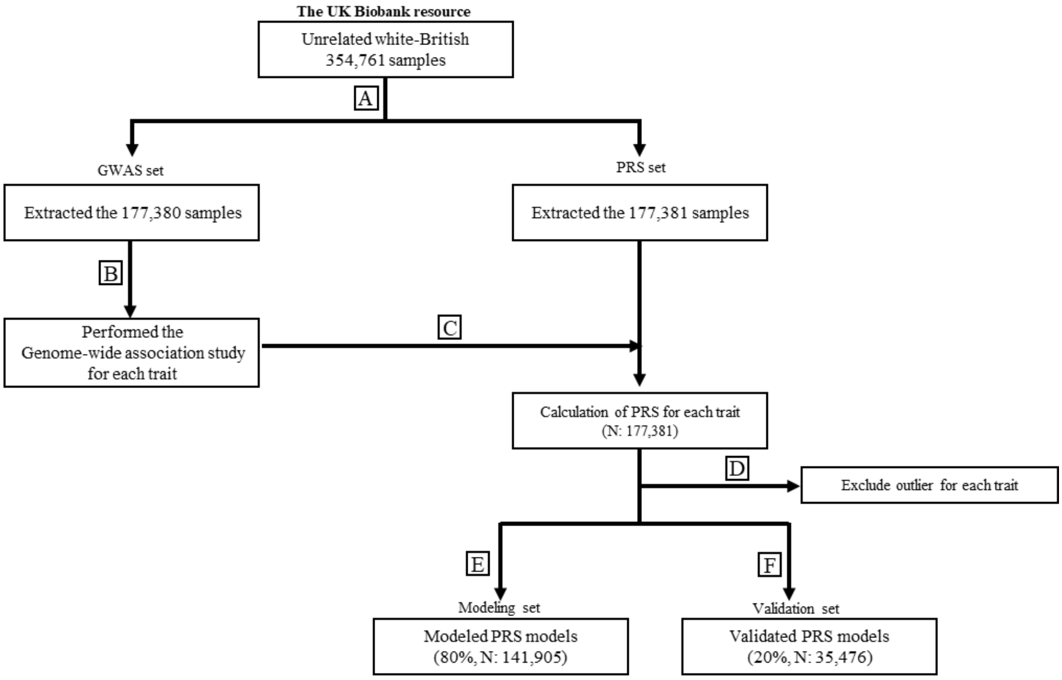

Supplementary Figure S1. Flow chart of the study design.

(A) We divided the 354,761 unrelated white-British samples in half.

(B) We performed the genome-wide association study (GWAS) using linear regression for each trait in the GWAS set.

(C) We calculated individual PRSs for each trait in the PRS set based on these GWAS summary statistics.

(D) The outlier was excluded for each trait to reduce bias.

(E) Polygenic risk score models were constructed for each trait and heteroscedasticity tests were performed on the PRS models.

(F) The proportion of error by the PRS group was investigated to determine whether heteroscedasticity affects differences in the prediction accuracy of PRS models by PRS groups in the validation set.

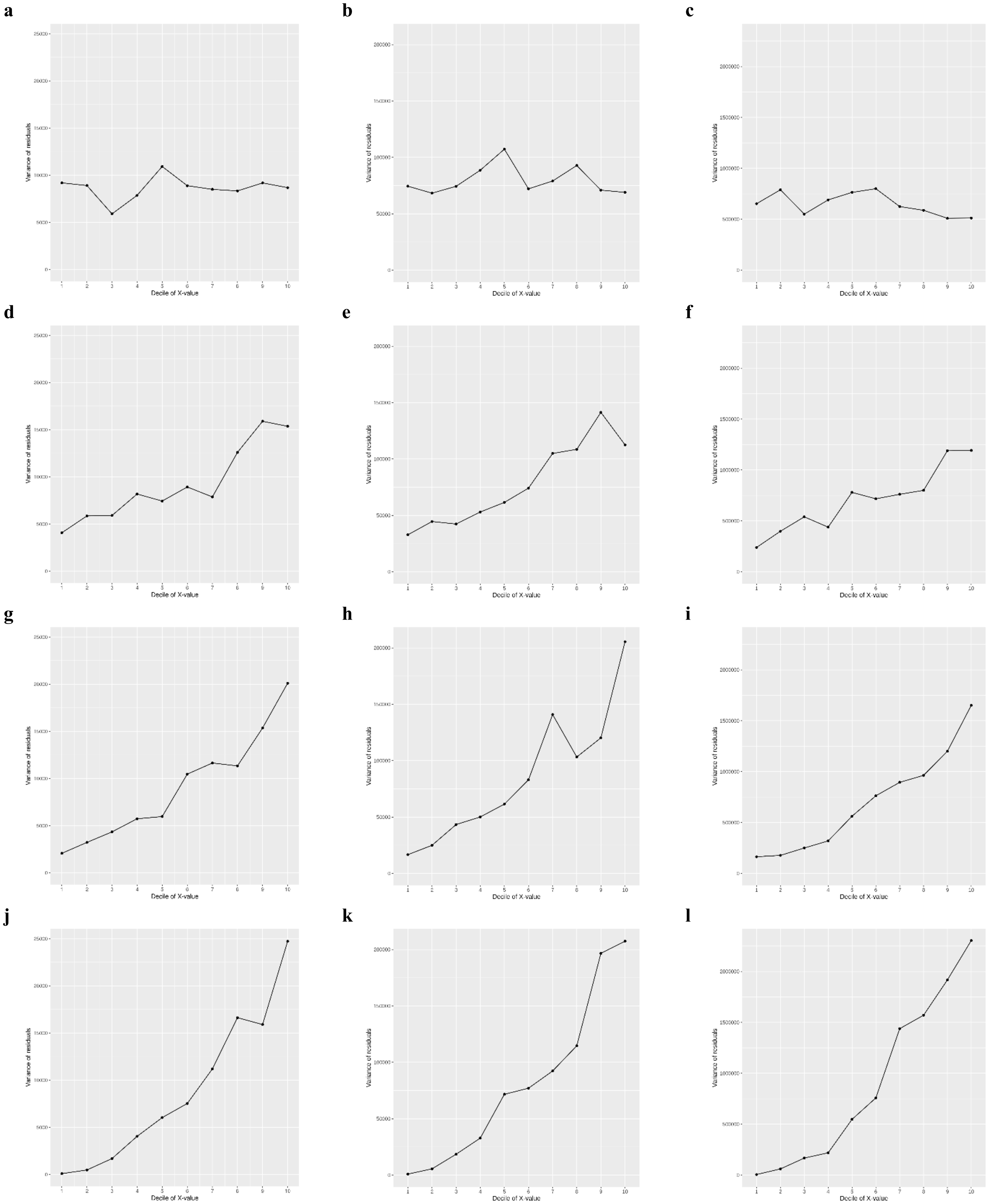

Supplementary Figure S2. Line graph of residuals variance by the decile group in 12 simulation data. Note: G1-G10 from left to right on the X-axis. Homoscedasticity (HS0) is a-c. Mild heteroscedasticity (HS1) is d-f. Moderate heteroscedasticity (HS2) is g-i. Severe heteroscedasticity is (HS3) is j-l. The R^2 value of a, d, g, and j is 0.9. The R^2 value of b, e, h, and k is 0.5. The R^2 value of c, f, i, and l is 0.1.

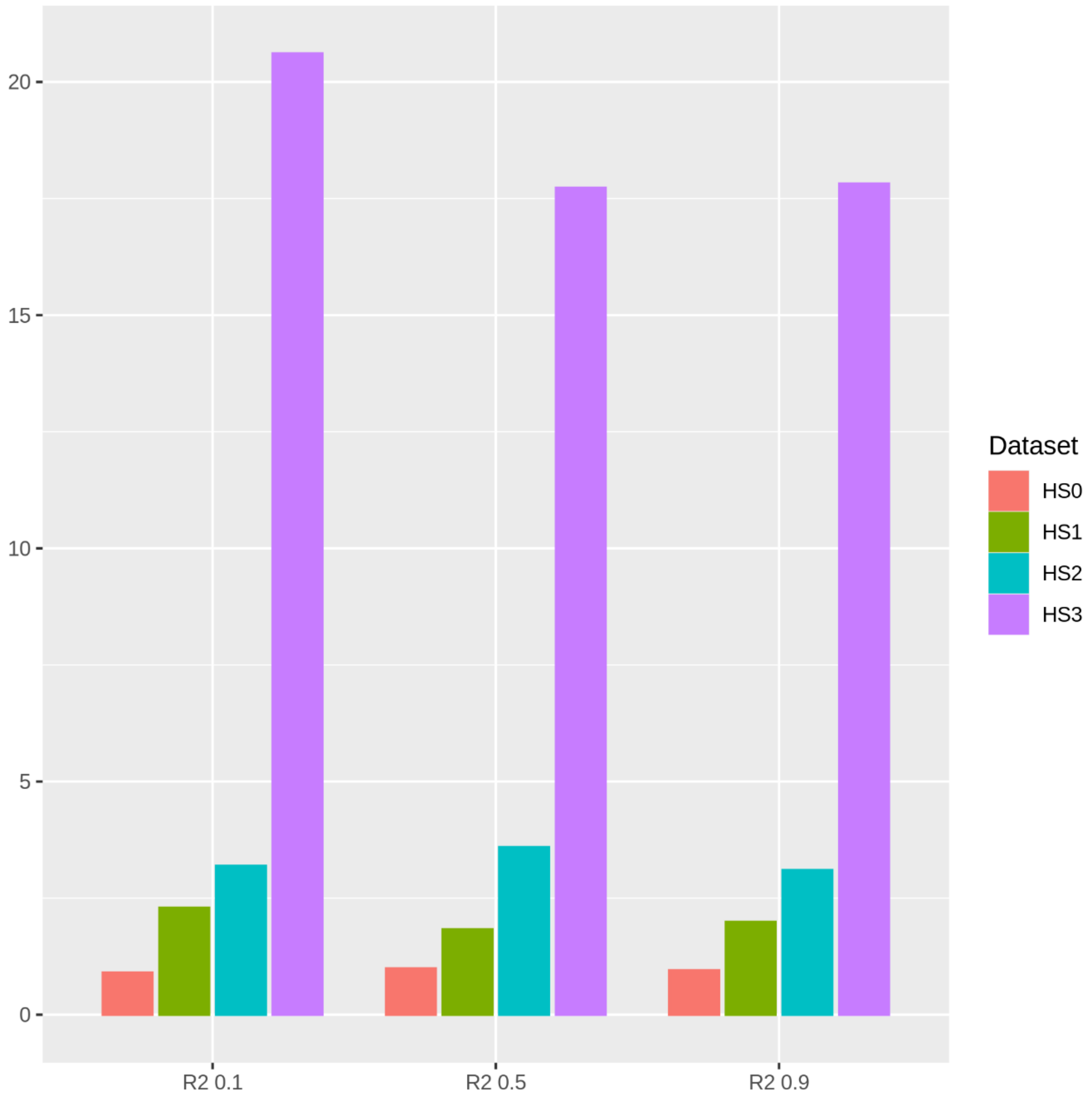

Supplementary Figure S3. Bar graph of the ratio of mean absolute residuals between G1 and G10 in 12 the simulation data (G1: 1st decile group of X-value, G10: 10th decile group of X-value).

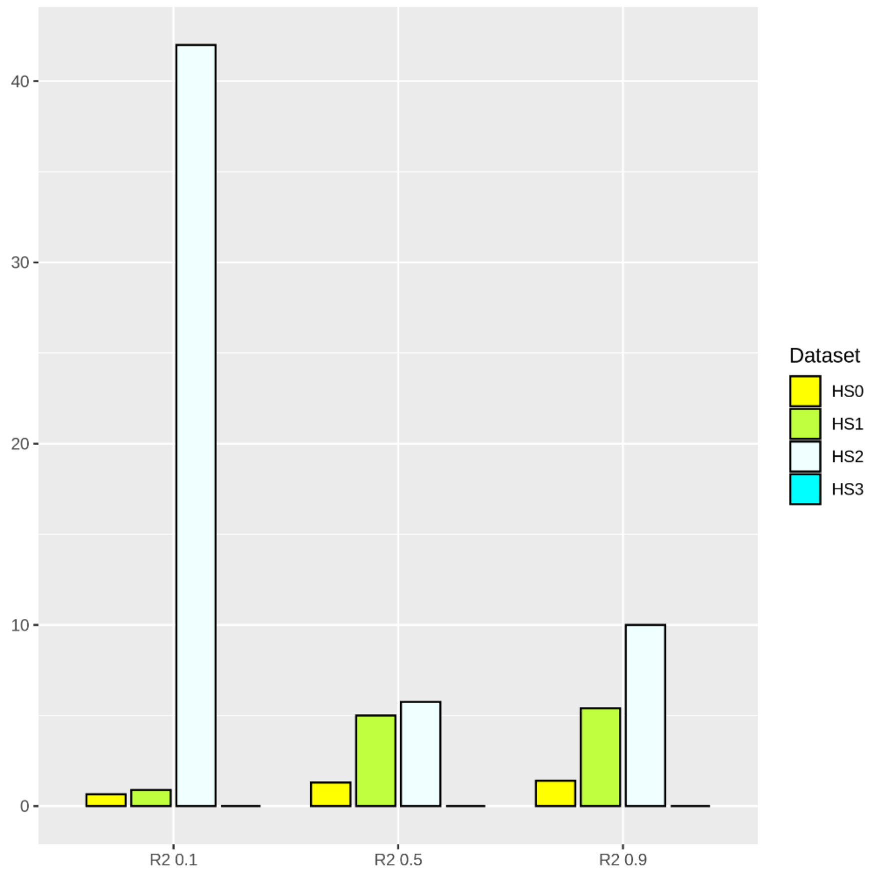

Supplementary Figure S4. Bar graph of ratio of error rate for G1 to G10 for 12 simulation data (G1: 1st decile group of X-value, G10: 10th decile group of X-value).

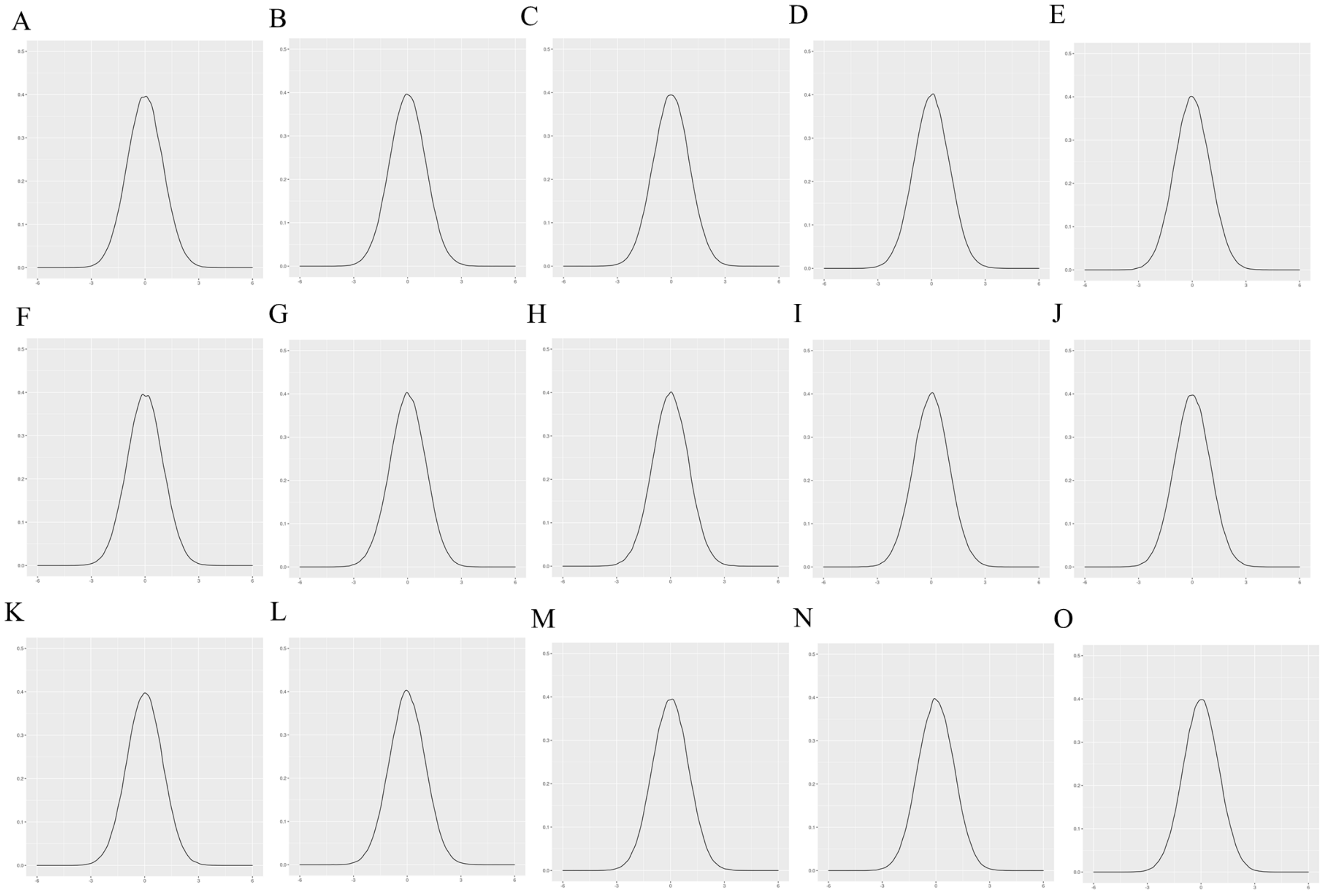

Supplementary Figure S5. Density plot of PRS. The X-axis is the standardized PRS. The Y-axis is the density according to the standardized PRS. (A) Alanine aminotransferase; ALT, (B) Alkaline phosphatase; ALP, (C) Aspartate aminotransferase; ALT, (D) Body mass index; BMI, (E) Cholesterol, (F) Creatinine, (G) Cystatin C, (H) Forced expiratory volume in one second and forced vital capacity ratio; FFR, (I) Height, (J) Phosphate, (K) Platelet count, (L) Red blood cell count; RBC, (M) Total protein; TP, (N) Triglycerides; TG, (O) Waist-to-hip ratio adjusted for BMI; WHR_{adjBMI}.

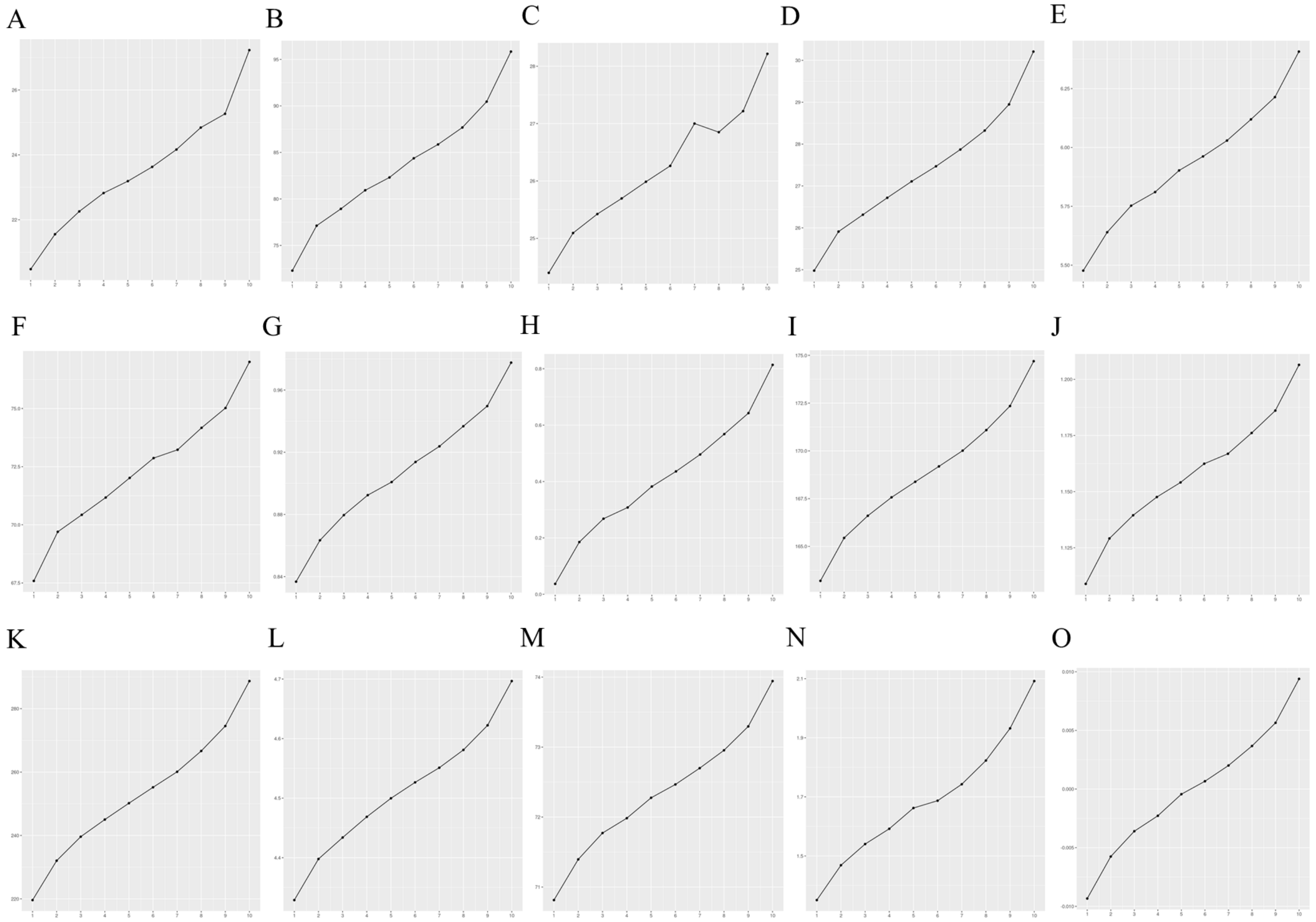

Supplementary Figure S6. Mean of 15 quantitative traits according to the decile of PRS groups. The X-axis is the decile of PRS groups. Note: G1-G10 from left to right on the X-axis. The Y-axis is the mean value of the trait in the decile of PRS groups. (A) Alanine aminotransferase; ALT, (B) Alkaline phosphatase; ALP, (C) Aspartate aminotransferase; ALT, (D) Body mass index; BMI, (E) Cholesterol, (F) Creatinine, (G) Cystatin C, (H) Forced expiratory volume in one second and forced vital capacity ratio; FFR, (I) Height, (J) Phosphate, (K) Platelet count, (L) Red blood cell count; RBC, (M) Total protein; TP, (N) Triglycerides; TG, (O) Waist-to-hip ratio adjusted for BMI; WHR_{adjBMI}.

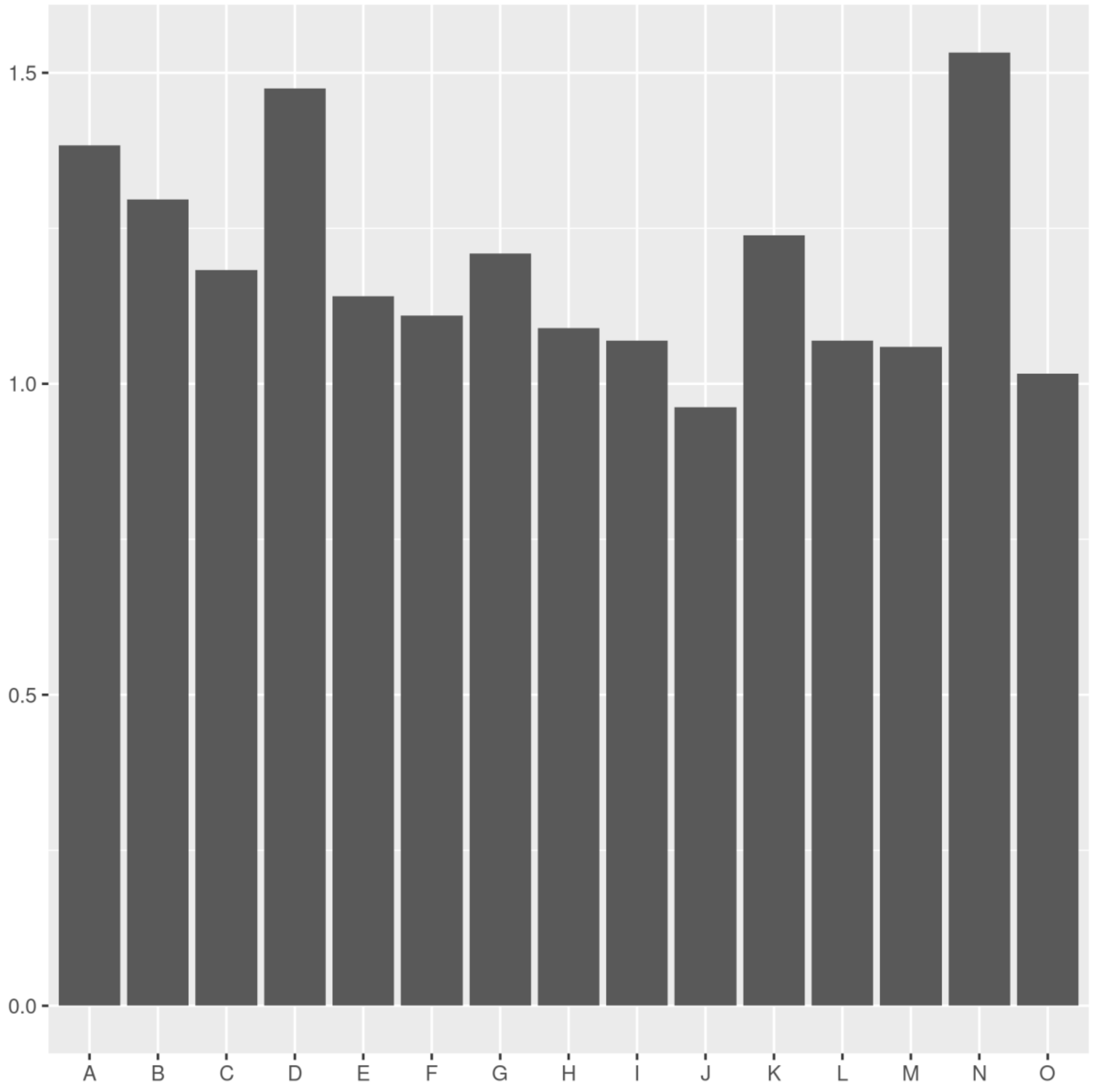

Supplementary Figure S7. Bar graph of the ratio of mean absolute residuals for G1 to G10 for 15 quantitative traits (G1: 1st decile group of PRS, G10: 10th decile group of PRS). (A) Alanine aminotransferase; ALT, (B) Alkaline phosphatase; ALP, (C) Aspartate aminotransferase; ALT, (D) Body mass index; BMI, (E) Cholesterol, (F) Creatinine, (G) Cystatin C, (H) Forced expiratory volume in one second and forced vital capacity ratio; FFR, (I) Height, (J) Phosphate, (K) Platelet count, (L) Red blood cell count; RBC, (M) Total protein; TP, (N) Triglycerides; TG, (O) Waist-to-hip ratio adjusted for BMI; WHR_{adjBMI}.

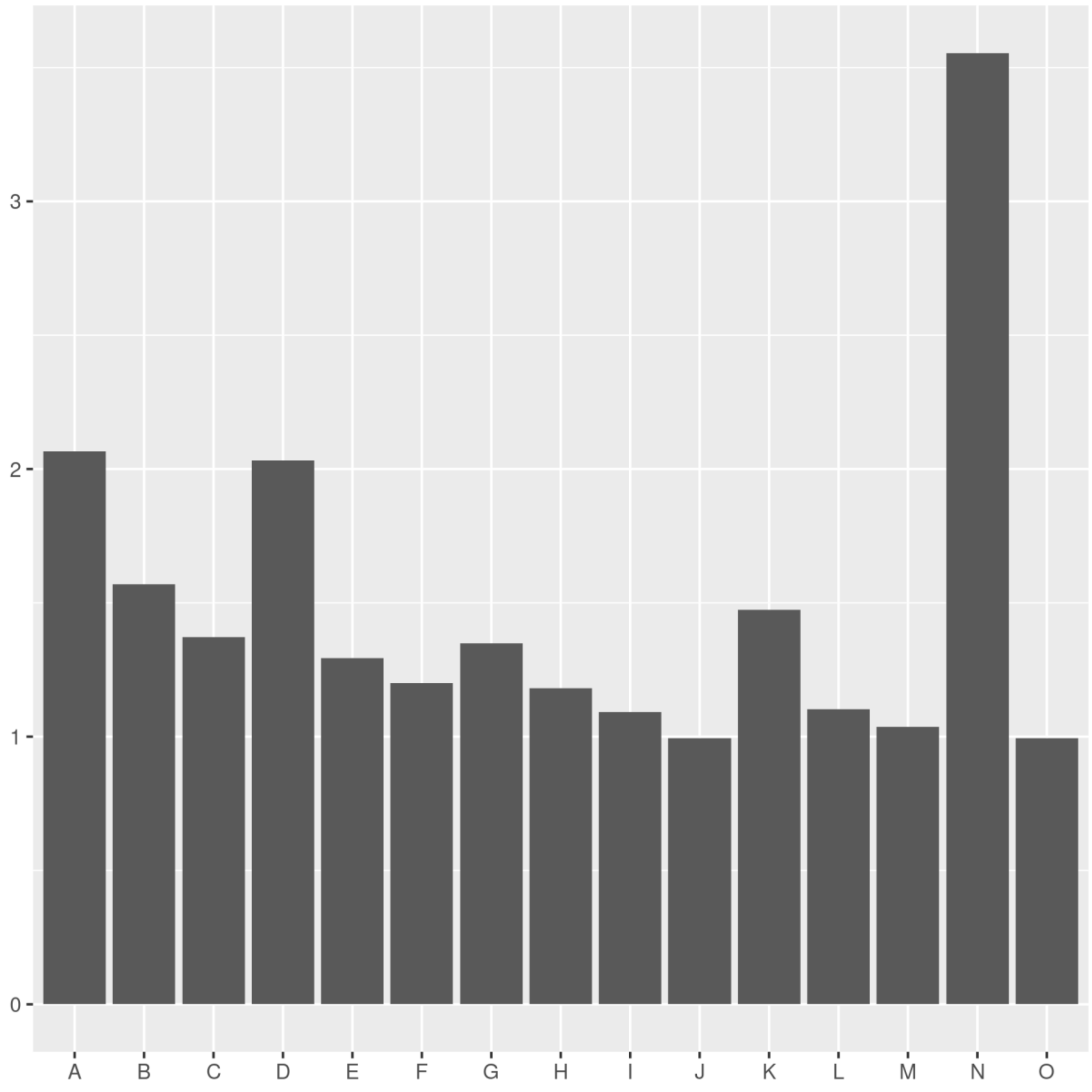

Supplementary Figure S8. Bar graph of ratio of error rate for G1 to G10 for 15 quantitative traits (G1: 1st decile group of PRS, G10: 10th decile group of PRS). (A) Alanine aminotransferase; ALT, (B) Alkaline phosphatase; ALP, (C) Aspartate aminotransferase; AST, (D) Body mass index; BMI, (E) Cholesterol, (F) Creatinine, (G) Cystatin C, (H) FEV1/FVC ratio Z-score; FFR, (I) Height, (J) Phosphate, (K) Platelet count, (L) Red blood cell count; RBC, (M) Total protein; TP, (N) Triglycerides; TG, (O) Waist-to-hip ratio adjusted for BMI
